# Coronavirus Disease-2019 Survival in Mexico: A Cohort Study on the Interaction of the Associated Factors

**DOI:** 10.3389/fpubh.2021.660114

**Published:** 2021-07-27

**Authors:** Horacio Márquez-González, Jorge F. Méndez-Galván, Alfonso Reyes-López, Miguel Klünder-Klünder, Rodolfo Jiménez-Juárez, Juan Garduño-Espinosa, Fortino Solórzano-Santos

**Affiliations:** ^1^Department of Clinical Research, Hospital Infantil de México Federico Gómez, Mexico City, Mexico; ^2^Centre for Research in Emerging Diseases, Hospital Infantil de México Federico Gómez, Mexico City, Mexico; ^3^Centre for Health Economics, Hospital Infantil de México Federico Gómez, Mexico City, Mexico; ^4^Research Management, Hospital Infantil de México Federico Gómez, Mexico City, Mexico; ^5^Clinical Infectious Disease Department, Hospital Infantil de México Federico Gómez, Mexico City, Mexico; ^6^Clinical Research Direction, Hospital Infantil de México Federico Gómez, Mexico City, Mexico; ^7^Infectious Diseases Research Department, Hospital Infantil de México Federico GómezMéxico Federico Gómez, Mexico City, Mexico

**Keywords:** Mexico, SARS-CoV-2, survival analysis, cohort study, comorbidity, COVID-19 outbreak

## Abstract

The pandemic caused by the new coronavirus Severe Acute Respiratory Syndrome Coronavirus-2 (SARS-CoV-2) is currently affecting more than 200 countries. The most lethal clinical presentation is respiratory insufficiency, requiring attention in intensive care units (ICU). The most susceptible people are over 60 years old with comorbidities. The health systems organization may represent a transcendental role in survival.

**Objective:** To analyze the correlation of sociodemographic factors, comorbidities and health system organization variables with survival in cases infected by SARS-CoV-2 during the first 7 months of the pandemic in Mexico.

**Methods:** The cohort study was performed in a health system public basis from March 1st to September 30th, 2020. The included subjects were positive for the SARS-CoV-2 test, and the target variable was mortality in 60 days. The risk variables studied were: age, sex, geographic distribution, comorbidities, health system, hospitalization, and access to ICU. Bivariate statistics (*X*^2^-test), calculation of fatality rates, survival analyses and adjustment of confusing variables with Cox proportional-hazards were performed.

**Results:** A total of 753,090 subjects were analyzed, of which the 52% were men. There were 78,492 deaths (10.3% of general fatality and 43% inpatient). The variables associated with a higher risk of hospital mortality were age (from 60 years onwards), care in public sectors, geographic areas with higher numbers of infection and endotracheal intubation without management in the ICU.

**Conclusions:** The variables associated with a lower survival in cases affected by SARS-CoV-2 were age, comorbidities, and respiratory insufficiency (with endotracheal intubation without care in the ICU). Additionally, an interaction was observed between the geographic location and health sector where they were treated.

## Introduction

The Severe Acute Respiratory Syndrome Coronavirus-2 (SARS-CoV-2) outbreak has been declared as a pandemic by the World Health Organization. The first case of this disease in Mexico was reported on April 28th, 2020. Until April 1, 2021, 131,435,555 cases of Coronavirus Disease-2019 (COVID-19) were registered worldwide, with a global fatality rate of 2.17% (1). At that time, 2,443,755 cases and 204,147 deaths were confirmed in Mexico, with a fatality rate of 9% (2). The disease is highly contagious, and although fatality remains low, there is a constant increase in the number of new cases in Mexico with a higher fatality rate than that observed globally (1).

Several changes in some epidemiological indicators have been observed worldwide: according to the initial findings in China, it was indicated that older adults presented more severe symptoms. In Europe, the population over 60 years old was the most affected, which coincide with being the community with the highest life expectancy in the world (3–6). In Latin-America, COVID-19 is also frequently observed in the population under 60 years of age, whereas in Brazil, 47% of cases occur between 20 and 59 years of age, and this was associated with the presence of comorbidities such as obesity, diabetes and hypertension, which are frequent at early ages (6–9). There is no specific treatment so far; a decrease in SARS-CoV-2 fatality cases was achieved with an appropriate care, including prompt hospitalization, mechanical ventilation, and attention in an intensive care unit (10, 11).

The hospital reconversion in Mexico and other countries has enabled an increase in resources (more hospitals, ventilators, and intensive care units (ICU) for patients with acute and severe COVID-19. According to daily reports in Mexico, there was an increase in the number of ICU beds or “beds with ventilators” from 2,446 to 11,346 over a 10-month period (12). However, the changes in fatality rates were not substantial (12). A hypothesis derived from early assessments showed that comorbidities in the Mexican population have a negative impact on survival, especially in cases of diabetes, arterial hypertension, and obesity (13, 14). In addition, there are other related conditions to death, such as the hospital's number and the health services quality that are variously distributed in the country. These variables must be weighted to identify the riskiest conditions. The experience in the results presented in Mexico may be helpful for other countries with similar social, economic and health system conditions, for a better chance in their public health strategies.

The aim of this study was to analyze the survival of confirmed cases with SARS-CoV-2 in the first 7 months of the national pandemic, assessing the impact of different factors as age, sex, comorbidities, healthcare system organization, medical unit geographic location, modality of care received, and access to ICU.

## Materials and Methods

The data used was from the open database of the Viral Respiratory Disease Epidemiological Surveillance Systems published daily by the Ministry of Health of Mexico (2). Among the variables available in the databases, the type of institution of the National Health System that provided care, federative entity where the medical unit was located, type of care (ambulatory, hospitalized), date of admission, gender, age, place of residence, date of symptoms onset, intubated or inpatient in ICU, presence of comorbidities, smoking history, pregnancy, and date of death if occurred were included. The confirmation of SARS-CoV-2 infection was performed by real-time polymerase chain reaction technique in certified laboratories by the National Institute of Epidemiological Reference.

A cohort of patients with positive SARS-CoV-2 test result was integrated from February 28th to September 30th, 2020. The day 0 of every patient was considered as the disease confirmation date and tracking was done till day 60 or the date death. The exposition variables were age (categorized in groups: <2, 2.1–5, 6–10, 11–20, 21–30, 31–40, 41–50, 51–60, 61–70, 71–80, and >80 years), sex, federative entity (32 States of Mexico). The following comorbidities were registered: obesity, diabetes, arterial hypertension, asthma or chronic obstructive pulmonary disease (COPD), immunosuppression and other risk factors such as smoking and pregnancy. Main health institutions of the country were analyzed: private institutions, Secretaría de Salud (SS), Instituto Mexicano de Seguridad Social (IMSS), Instituto de Seguridad y Servicios Sociales de los Trabajadores del Estado (ISSSTE), Petroleos de México (PEMEX), Secretaría de la Marina (SEMAR), Secretaría de la Defensa Nacional (SEDENA), State Hospitals, and the group of “other public” that assist <2% of the population. The outcome variables also included were: hospitalization, pneumonia, invasive mechanical ventilation (IMV), and admission to ICU. The main outcome variable was survival till 60 days.

### Statistical Analysis

Descriptive statistical analyses of the variables of interest were performed by calculating the relative frequencies, and independent hypothesis tests (*X*^2^) were used to assess correlations between qualitative variables. The softened risk density in patients hospitalized vs. those in ambulatory care was analyzed using Nelson–Aalen estimation (Supplementary Material). The analyses of survival were performed using Kaplan–Meier method with the log rank test. Finally, different models of Cox regression were constructed to evaluate the effect of the different clinical, demographic, and socio-economic factors on the period of death. The criteria for introducing the model were *p*-value < 0.05 or biological plausibility. Finally, three different models were built: the total population, hospitalized patients, and the ICU. Internal validation of each model was performed by calculating each patient's probability in the cohort with the formula, $\lambda \left(t\right)=\lambda_{0}\left(t\right)\exp\left(\beta^{T}X\right)$, and estimating the value of the area under the curve (AUC) with the death variable (15). The entire analysis was performed using the Stata 16.1 version.

## Results

A total of 1,735,597 observations were included in the original basis, of which were eliminated for analysis 893,324 (51.5%) who corresponded to patients with negative SARS-CoV-2 test, and 89,186 (5.1%) individuals whose results were not available.

**Figure 1:**
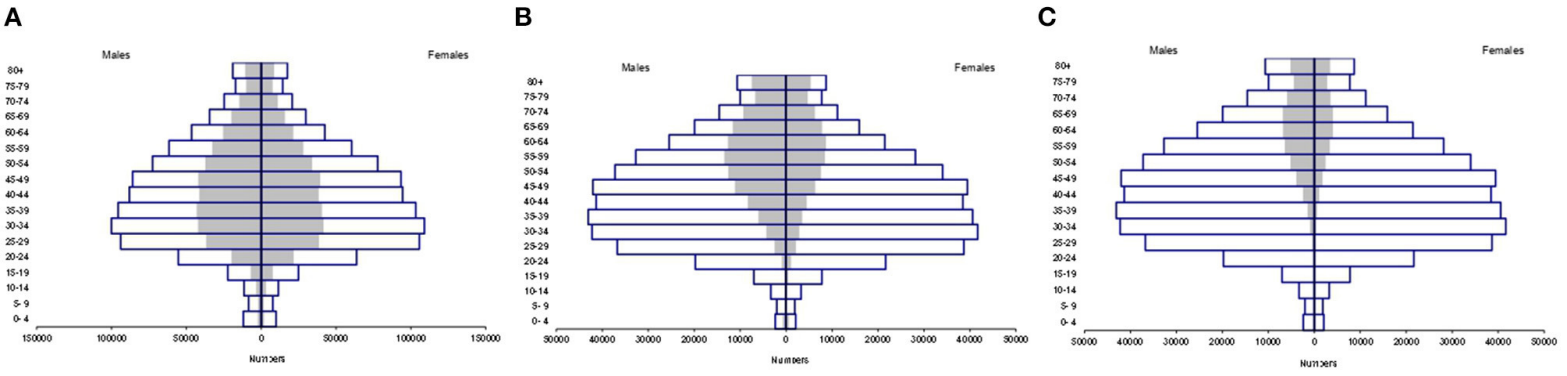
Distribution according to age group and sex, in Mexico. **(A)** Distribution of suspected cases (white bar) and positive cases (gray bar) according to age group and sex, in Mexico. **(B)** Distribution of positive cases (white bar) and hospitalized cases (gray bar) according to age group and sex, in Mexico. **(C)** Distribution of patients who died from SARS Cov-2 by age group and sex in Mexico. Positive cases (white bar) and deaths (gray bar).

Analysis was made with 753,090 patients with positive SARS-CoV-2 test, 52% were males, and >55% of the population was grouped between 20 and 59 years old (Figure 1A). Male sex was predominant in all age groups requiring hospitalization. Both men (70.2%) and women (61.5%) aged >80 years required a higher proportion of hospitalizations. The lowest hospitalization proportion was observed in younger people (15–24 years old group), Figure 1B.

A total of 78,534 deaths were registered. The lowest fatality rate was observed in people <18 years of age (0.86%), followed in ascending order by patients of age groups 19–40 (7.03%), 40–60 (35.9%), and >60 (56.1%), the male population being the most predominant (Figure 1C).

Patients <18 years of age had a lower percentage of comorbidities, and 14% of infected children had one comorbidity. The comorbidities observed in the descending order of frequency were obesity (3.7%), asthma (3.7%), immunosuppression (2.9%), diabetes mellitus (0.7%), arterial hypertension (0.7%), and chronic renal failure (0.7%). The greater comorbidities frequencies were observed in children of 11–18 years old; obesity (6%), asthma (4%), and immunosuppression (1.5%) were highlighted. It must be noted that 4.7% of infected people <18 years of age were obese and represented 0.8% of the total obese population infected with SARS-CoV-2 (Table 1).

**Table 1:**
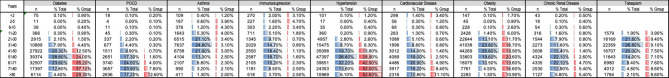
Comparison of presentation of comorbidities according to age in patients with SARS Cov-2.

*COPD, Chronic Obstructive Pulmonary Disease*.

The principal comorbidities observed among people aged ≥19 years included arterial hypertension (20%) and obesity (19%) followed by diabetes mellitus (16%), asthma (2.6%), chronic renal failure (1.9%), and immunosuppression (1%) in the descending order of frequency. The largest number of patients with some comorbidity is grouped between 19 to 60 years of age (Table 1).

A significant statistical difference was observed between the proportions of comorbidities in the group of non-surviving and surviving patients (Table 2). Higher fatality rates could be found in the age group of >60 years. Further, the high frequency of comorbidities in the age group of 40–60 years is of concern.

**Table 2:** Differences between survivors and non-survivors with SARS CoV-2.

**Variables**	**Survival**		**No survival**		***p*-value**
	***n***	**%**	***n***	**%**	
**Sex**
Male	340,076	50.40%	50,340	64.10%	<0.0001
Female	334,244	49.60%	28,152	35.90%	
**Group Age**
<2 years	2,383	0.40%	121	0.20%	<0.0001
2.1–5.9	2,557	0.40%	33	0.00%	
6–9.9	4,255	0.60%	39	0.00%	
10–18.9	15,628	2.30%	102	0.10%	
19– <40	284,456	42.20%	4,337	5.50%	
40– <50	151,988	22.50%	9,252	11.80%	
50– <60	114,663	17.00%	17,446	22.20%	
60– <70	60,976	9.00%	21,766	27.70%	
70–79.9	26,723	4.00%	16,741	21.30%	
80+	10,691	1.60%	8,655	11.00%	
**Type**
Ambulatory	564,547	83.70%	8,977	11.40%	<0.0001
Hospitalization	109,773	16.30%	69,515	88.60%	
**Comorbidities**
Diabetes	85,614	12.70%	30,096	38.30%	<0.0001
COPD	7,085	1.10%	3,813	4.90%	<0.0001
Asthma	17,889	2.70%	1,578	2.00%	<0.0001
Immunocompression	6,159	0.90%	1,935	2.50%	<0.0001
Hypertension	109,956	16.30%	35,221	44.90%	<0.0001
Cardiovascular disease	10,572	1.60%	4,175	5.30%	<0.0001
Obesity	116,071	17.20%	19,182	24.40%	<0.0001
Renal chronic disease	8,635	1.30%	5,529	7.00%	<0.0001
Smoking	48,622	7.20%	6,264	8.00%	<0.0001
**Outcomes**
Pneumonia	79,584	11.80%	58,078	74.00%	<0.0001
Invasive mechanical ventilation	5,898	0.90%	25,539	32.50%	<0.0001
Intensive care unit	7,514	1.10%	7,890	10.10%	<0.0001

*The value of p was calculated by X^2^ test*.

According to geographical distribution (Table 3), a large number of positive cases and deaths were concentrated in Mexico City (CDMX) and Mexico State (EDOMEX), which are states with the largest population in our country. The national case fatality rate was 10.4%, whereas that in CDMX and EDOMEX was 7.98 and 16.2%, respectively.

**Table 3:**
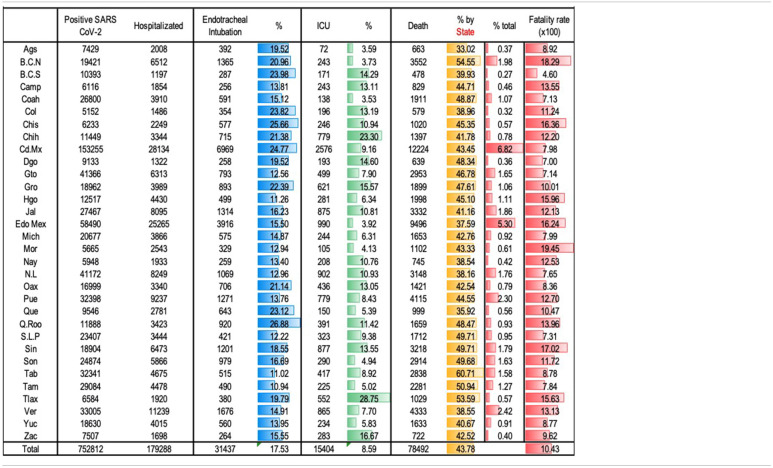
Hospitalization, ICU care, endotracheal intubation, and fatality by State..

*Ags, Aguascalientes; B.C.N, Baja California Norte; B.C.S, Baja California Sur; Camp, Campeche; Coah, Coahuila; Col, Colima; Chis, Chiapas; Chih, Chihuahua; Cd.Mx, Ciudad de México; Dgo, Durango; Gto, Guanajuato; Hgo, Hidalgo; Jal, Jalisco; Edo Mex, Estado de México; Mich, Michoacán; Mor, Morelos; Nay, Nayarit; N.L, Nuevo León; Oax, Oaxaca; Pue, Puebla; Que, Querétaro; Q.Roo, Quintana Roo; S.L.P, San Luis Potosí; Sin, Sinaloa; Son, Sonora; Tab, Tabasco; Tam, Tamaulipas; Tlax, Tlaxcala; Ver, Veracruz; Yuc, Yucatán; Zac, Zacatecas*.

Some entities with lower population density showed greater case fatality rates, such as the States of Morelos, Baja California, Sinaloa, Chiapas, and Hidalgo. In other states, population density was not associated with case fatality rate.

23.8% of positive SARS-Cov-2 patients required hospitalization of which 17.5% received endotracheal intubation and 8.6% were admitted in an ICU. The proportion of patients who required endotracheal intubation and who could be attended to in an ICU was variable in different states; however, the case fatality rate was higher in those states with a higher rate of intubated patients with lower admissions in ICU than others.

In study period 23.8% (179,288) of the patients with SARS-CoV-2 infection were hospitalized (Table 4); among these, 76.8% had pneumonia as the main diagnosis, 20.1% required intubation and 8.6% was inpatient in an ICU. Nationally, 89.3% of the cases were attended in three institutions: IMSS (48.1%), SS (33.6%) and ISSSTE (7.6%). The institutional case fatality rate (CFR) was 43.7%. Among the patients admitted to IMSS, 30% had pneumonia, 10.9% who were intubated required IMV, and 2% were attended to in an ICU. Among patients admitted at SS, 30.8% had pneumonia, 6.2% required IMV, and 4.8% were treated in an ICU. Private hospitals attended to 2.1% of hospitalized patients with COVID-19. Among these, 22% required IMV and 27% were admitted in an ICU. The CFRs for IMSS, SS, and private hospitals were 50.7, 39.2, and 19.4%, respectively.

**Table 4:**
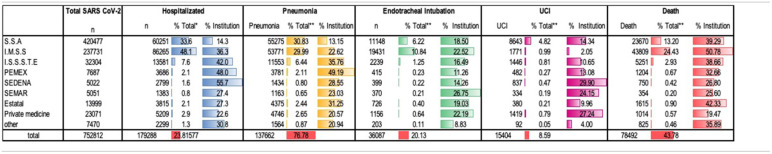
Outcomes according to the Health System.

* *n/hospitalizated*.

** *n/hospitalizated by institution*.

*SS, Secretaría de Salud; IMSS, Instituto Mexicano del Seguro Social; ISSSTE, Instituto de Seguridad y Servicios Sociales de los Trabajadores del Estado; SEDENA, Secretaría de la Defensa Nacional; SEMAR, Secretaría de Marina*.

Other's health system hospitals where ICU attention was greater were SEDENA (29.9%), private hospitals (27.2%), and SEMAR (24.1%). These units had lower CFR, 14.9, 4.3, and 7.1%, respectively.

**Figure 2:**
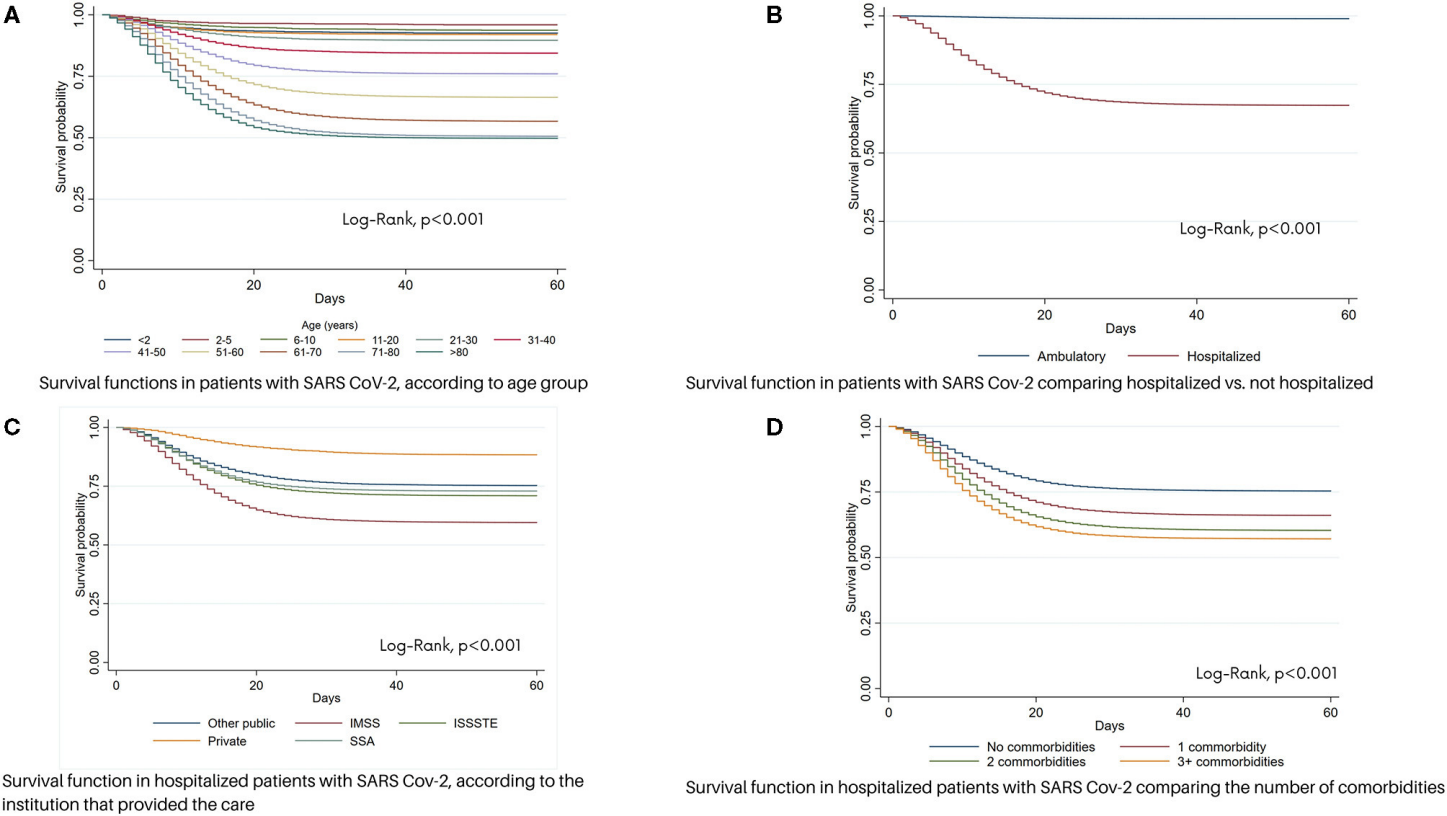
Survival function in patients with SARS Cov-2 in México. **(A)** Survival functions in patients with SARS Cov-2, according to age group. **(B)** Survival functions in patients with SARS Cov-2 comparing hospitalized vs. non- hospitalized. **(C)** Survival function in hospitalized patients with SARS Cov-2, according to the institution that provided the care. **(D)** Survival function in hospitalized patients with SARS Cov-2 comparing the number of comorbidities.

In non-hospitalized patients, the survival at 60 days was 95.4%; in contrast survival in hospitalized patients was 75%, with an survival average of 11 days (*p* < 0.05) (Figure 2A). In addition, in hospitalized patients, there was a direct association between age and survival (*p* < 0.05) (Figure 2B). Patient survival was higher (average: 33 days) among those hospitalized in private than in public institutions. Lower survival (average: 10 days) was observed in patients admitted to IMSS (Figure 2C).

On comparing the probability of survival based on the number of comorbidities, patients with three or more comorbidities were found to have a lower probability of survival (Figure 2D).

The adjustment of the effect of the variables with mortality was performed using three Cox proportional models (Table 5). The individual probability of outcome presentation by each subject according to each model was calculated. In the first prognostic model, the mortality of all subjects was explained, including all variables with a *p*-value < 0.05 (sex, age group, health service, comorbidities, the need for hospitalization, and pneumonia), resulting in 10 associated variables, two related to the health system (private practice with protective effect), five comorbidities and their sum, pneumonia development, and need for hospitalization. The summed probability of the model showed an area under the curve (AUC) of 0.86.

**Table 5:** Proportional Cox Regression for prognostic mortality in patients with SARS CoV-2.

**(A) Prognostic variables associated in all population**					
	**β**	**HR**	**95% CI**		***p*-value**
Social Security	1.221	3.391	3.304	3.481	0.0001
Private Hospital	−0.221	0.802	0.721	0.893	0.001
Diabetes	0.654	1.924	1.823	2.031	0.001
COPD	0.424	1.528	1.426	1.636	0.001
Hypertension	0.736	2.088	1.977	2.206	0.001
Immunossupresion	0.117	1.124	1.034	1.221	0.02
Chronic Renal Disease	0.26	1.297	1.216	1.383	0.002
Comorbidities (number)	0.29	1.336	1.276	1.4	0.0001
Hospitalization	2.83	17	16.25	17.78	0.0001
Pneumonia	1.124	3.07	2.96	3.17	0.0001
AUC = 0.86					
**(B) Prognostic variables associated in patients hospitalizated**					
Age>60 years	0.586	1.796	1.22	2.644	0.003
Male	0.151	1.163	1.15	1.176	0.0001
Social Security	0.156	2.304	2.176	2.44	0.02
Private Hospital	−1.382	0.251	0.204	0.309	0.0001
Health minstery	0.285	1.33	1.212	1.46	0.0001
Asthma	0.107	1.113	1.034	1.198	0.004
Tabaquism	0.157	1.17	1.097	1.248	0.0001
Pregnancy	1.351	3.86	3.075	4.846	0.0001
ICU	0.054	1.055	1.009	1.103	0.018
Advanced mechanical ventilation	0.724	2.063	2.038	2.089	0.0001
ICU and AMV	−0.142	0.868	0.829	0.908	0.0001
Comorbidities (number)	0.123	1.2	1.1	1.3	0.0001
AUC = 0.78					
**(C) Prognostic variables associated in patients in ICU**					
Male	0.075	1.078	1.042	1.114	0.001
Age 60 years	1.942	6.974	6.041	8.051	0.0001
Social Security	0.988	2.686	1.058	6.819	0.0001
Private	−1.451	0.234	0.117	0.469	0.0001
Diabetes	0.091	1.095	1.059	1.133	0.0001
Immunossupresion	0.108	1.114	1.023	1.214	0.0001
Obesity	0.054	1.055	1.018	1.093	0.0001
Chronic Renal Disease	0.171	1.186	1.108	1.269	0.0001
Comorbidities (number)	0.233	1.3	1.2	1.4	0.0001

*AUC = 0.65; β, beta coefficient; CI, Confidence Interval; HR, Hazard Ratio*.

The second model variables (sex, age >60 years, type of health service, comorbidities, ICU, and IMV) were adjusted in hospitalized patients and demonstrated the association of 12 variables, which differed from model 1 and additionally included the following: age >60 years, male, asthma, smoking, and pregnancy. In terms of independent care in the ICU and AMV, the variables demonstrated an association with the risk of death; however, the combination of both offered a protective effect. The AUC value was determined as 0.78.

The last model was carried out in ICU patients, demonstrating a primary association between the type of health services and comorbidities. The AUC value was observed to be 0.65.

## Discussion

We have provided an analysis of the first 7 months from the start of the COVID-19 pandemic in Mexico. CFR has been considered higher for Mexico compared with many other countries. However, during the first 3 months of the pandemic, sampling of the people suspected to have SARS-Cov-2 virus was limited; “sentinel monitoring” was used (only one in every 10 suspected ambulatory cases and all hospitalized cases were sampled). Therefore, CFR denominator was underestimated (diagnosis confirmation bias). The number of samples gradually increased and included all clinically suspected patients. Only 14 tests per 1,000 people were carried out in Mexico, which was in contrast to that observed in a study carried out in Chile, where 130 PCR tests were carried out per 1,000 people, obtaining a fatality rate of 4.16% (16).

Considering the COVID-19 registered deaths around the world, Mexico is placed as the 3th country of the world with major number of deaths, behind the United States and Brazil (1). Age has been considered one of the most outstanding death risk factors in most countries of the world. The percentage of deaths in the first wave in people over 60 years of age in Italy and China were 96.5 and 81%, respectively. A total of 98% of the deceased were older than 50 years in England, 97.5% were older than 45 years in USA, and 56.2% were over 60 years of age in Mexico (5, 17, 18). It is of interest that 19.5% of the deceased individuals in Mexico were between 21 and 50 years of age, which corresponds to a young and economically active population. In Mexico, a mandatory lockdown was not imposed, and Mexican population with low and middle income, has no savings capacity; hence, people had the necessity to work, this factor could influence on the higher disease incidence and higher mortality rates observed in young people with risk factors for developing a serious disease (13, 14).

In our country infected patients aged <20 years represented 3.1% of the total infected population; the CFR was 0.12%, which was similar to that observed in Spain, Italy, Germany, China, and South Korea (19, 20). The number of infected children in Mexico has been strikingly higher than that in countries. Little is known about COVID-19 and comorbidities in children (21). In the infected Mexican population aged <18 years, the percentage of comorbidities was low (14%); obesity (4.7%) and diabetes mellitus (0.8%) were not factors with a high incidence, which was in contrast to that observed in the adult population. Comorbidities were most frequent in children aged 11–18 years. Most of the Mexican children did not have any comorbidity. A Saudi Arabian cohort, which included population aged <1 and >5 years, was observed to have a high rate of hospitalization, with a low percentage of comorbidities (22).

There was a relationship in mortality between the economic States conditions and the health services distribution; for example, difference in the fatality rate was twice higher in the Edo Mex than in the Cd Mx despite that Edo Mex population is twice as high (16,992,418 vs. 9,209,944 people) (23), but the gross domestic product is 45% lower in Edo Mex, a condition reflected in the provision of health services; in Cd Mx the number of third level hospitals is 3.5 higher (56 vs. 16 hospital centers) (24). The impact of sociodemographic conditions on mortality in Brazil was evaluated by Braga-Ribeiro et al., who found an association between less education, more household crowding, lower income, and a higher population concentration in subnormal areas; mortality was found to be four times higher in a population with a lower degree of education compared with that having a higher degree of education, showing that socio-economic inequity impacts fatality during this pandemic (25).

Nation-wide, the proportion of confirmed cases that required hospitalization was 23.8%, 17.5% were intubated and 8.6% were inpatient in an ICU and 43.8% of hospitalized patients died with a CFR of 10.4%. A total of 50% of patients requiring endotracheal intubation received management outside an ICU; fatality was higher in such individuals than in than those who received attention in an ICU. There was variation according to every state, for example, in Baja California, 33% of the confirmed cases were hospitalized and 20.9% of these were intubated, but only 3.7% were admitted in an ICU. In this state, the CFR was 18.3%. In contrast Chihuahua, where 29% of the cases were hospitalized, 23.3% were admitted to an ICU, and 21.4% received attention outside an ICU, the CFR was 12.2%. In the analysis by Health Institutions, the differences between the two institutions that serve the largest proportion of the national population stand out; 10% of admitted patients required endotracheal intubation and 2% were treated in an ICU in the IMSS, which had a fatality rate of 19%, whereas 15% of admitted patients required endotracheal intubation and 14% were treated in an ICU at the SSA, which had a 6% fatality rate. Despite the fact that with health system policies there was an increase in the number of hospital beds and ventilators, the fatality rate in critically ill patients requiring endotracheal intubation was high, as observed in a previous study. This suggests that the quality of care was inadequate due to the lack of expertise of the medical and paramedical groups in ventilatory management and critical care medicine; however, expertise has improved as the pandemic progresses. Having only a sufficient number of beds with ventilators does not ensure optimal care or a better prognosis for patients with acute respiratory distress syndrome due to SARS-CoV-2. In this analysis, critical patients cared in ICU were not observed to be at higher risk of death vs. those who were intubated outside of an ICU, revealing infrastructure and specialized staff importance in care of such patients.

In this study due to the database characteristics, the period of disease progression and clinical conditions at the time of requesting medical care cannot be correlated, as well as the time interval between endotracheal intubation and displacement to ICU. In two studies in Mexico on patients receiving ICU care, the average time between the onset of symptoms and the inpatient hospitalization in ICU was 7 days (interquartile range 4.5–9), the mean of days from the presentation of symptoms to admission was 4.3 ± 3.4 days; from the admission to death was 5.9 ± 4.9 days; and from the presentation of symptoms to death was 10.1 ± 5.5 days (12, 13). There were 4.6% of non-hospitalized patients who died, which probably reflects health system problems during hospital service saturation and the low capacity of patients to recognize the severity of their condition and visit the hospital in a timely manner. The information collected however, was insufficient to delve into this topic.

The national database has been analyzed in other publications (13, 26). The three prognostic models presented in this work analyze the effect that risk variables have in different scenarios, particularly in patients in ICU; the analysis of Ñamendys Silva is consistent (12), who analyzed the decision made by the health authorities in Mexico during the second wave of infections, in December 2020, when the number of beds with a ventilator increased by 4.7 times (from 2,446 to 11,634) in hospital areas not equipped with intensive care, consequently, mortality was 12% higher, probably explained by human resources and the equipment had a lower quality of care. A recently published analysis in the pediatric population demonstrated the relationship among age, the clinical presentation with intubation, and the need for intubation as the variables associated with mortality (27).

Some limitations of this study include the following: (a) As each hospital center feeds the database and the results are issued as cases are added, reports on the outcomes are subject to the data being updated. Hence, it is possible that the population at cut-time may be underrated (follow-up bias); (b) Variables that assess the presence of comorbidities were obtained from questioning, and because the operational variables were not defined, a bias is highly probable (misclassification bias); (c) As hospitals followed a conversion strategy by using certain hospital areas as intensive care units, it is possible that the patients treated at this sites have been registered as intubated without ICU (registration bias); and (d) the construction of predictive models, using the PRISMA recommendations, shows that the variables analyzed could be insufficient to explain mortality fully. It is necessary to consider that, in the context of hospitalized patients, multiple variables that could be associated are involved (reporting bias).

## Conclusion

In Mexico, mortality from SARS-CoV-2 was found to be associated with age and a history of comorbidities. The provision of services in the public sector is associated with mortality due to the relationship between IMV and access to intensive care areas.

## Data Availability Statement

The datasets presented in this study can be found in online repositories. The names of the repository/repositories and accession number(s) can be found in the article/Supplementary Material.

## Ethics Statement

Ethical review and approval was not required for the study on human participants in accordance with the local legislation and institutional requirements. Written informed consent was not provided because Research subjects were obtained from the public database of the Secretariat of Health of Mexico. Access to the identity of the research subjects is not possible.

## Author Contributions

All authors contributed to the article and approved the submitted version. Analysis: HM-G, AR-L, MK-K, and FS-S. Manuscript writing: HM-G, JFM-G, MK-K, RJ-J, JG-E, and FS-S.

## Conflict of Interest

The authors declare that the research was conducted in the absence of any commercial or financial relationships that could be construed as a potential conflict of interest.

## Publisher's Note

All claims expressed in this article are solely those of the authors and do not necessarily represent those of their affiliated organizations, or those of the publisher, the editors and the reviewers. Any product that may be evaluated in this article, or claim that may be made by its manufacturer, is not guaranteed or endorsed by the publisher.

## Abbreviations

CDMX: City of Mexico
COVID-19: Coronavirus Disease-2019
EDOMEX: State of Mexico
ICU: intensive care unit
IMSS: Instituto Mexicano de Seguridad Social
IMV: invasive mechanical ventilation
ISSSTE: Instituto de Seguridad y Servicios Sociales de los Trabajadores del Estado
PEMEX: Petroleos de México
SARS-CoV-2: Severe Acute Respiratory Syndrome Coronavirus-2
SEDENA: Secretaría de la Defensa Nacional
SEMAR: Secretaría de la Marina
SS: Secretaría de Salud.
