# Corrigendum: Coronavirus Disease-2019 Survival in Mexico: A Cohort Study on the Interaction of the Associated Factors

**DOI:** 10.3389/fpubh.2021.754703

**Published:** 2021-09-29

**Authors:** Horacio Márquez-González, Jorge F. Méndez-Galván, Alfonso Reyes-López, Miguel Klünder-Klünder, Rodolfo Jiménez-Juárez, Juan Garduño-Espinosa, Fortino Solórzano-Santos

**Affiliations:** ^1^Department of Clinical Research, Hospital Infantil de México Federico Gómez, Mexico City, Mexico; ^2^Centre for Research in Emerging Diseases, Hospital Infantil de México Federico Gómez, Mexico City, Mexico; ^3^Centre for Health Economics, Hospital Infantil de México Federico Gómez, Mexico City, Mexico; ^4^Research Management, Hospital Infantil de México Federico Gómez, Mexico City, Mexico; ^5^Clinical Infectious Disease Department, Hospital Infantil de México Federico Gómez, Mexico City, Mexico; ^6^Clinical Research Direction, Hospital Infantil de México Federico Gómez, Mexico City, Mexico; ^7^Infectious Diseases Research Department, Hospital Infantil de México Federico GómezMéxico Federico Gómez, Mexico City, Mexico

**Keywords:** Mexico, SARS-CoV-2, survival analysis, cohort study, comorbidity, COVID-19 outbreak

In the original article, there were some errors. “Comorbidities” was misspelled. All instances of the incorrect spelling should be corrected to read “comorbidities”.

The acronym for chronic obstructive pulmonary disease was incorrectly spelled. This should be COPD.

In the original articles, there were some mistakes in Table 2.

The section “Taquism” should be named “Smoking”. The row “Taquism” in the “Outcomes” section was deleted. “Immunossupression” should read “Immunocompression”.

The corrected table appears below.

**Table 2:** Differences between survivors and non-survivors with SARS CoV-2.

**Variables**	**Survival**		**No survival**		***p*-value**
	***n***	**%**	***n***	**%**	
**Sex**
Male	340,076	50.40%	50,340	64.10%	<0.0001
Female	334,244	49.60%	28,152	35.90%	
**Group Age**
<2 years	2,383	0.40%	121	0.20%	<0.0001
2.1–5.9	2,557	0.40%	33	0.00%	
6–9.9	4,255	0.60%	39	0.00%	
10–18.9	15,628	2.30%	102	0.10%	
19– <40	284,456	42.20%	4,337	5.50%	
40– <50	151,988	22.50%	9,252	11.80%	
50– <60	114,663	17.00%	17,446	22.20%	
60– <70	60,976	9.00%	21,766	27.70%	
70–79.9	26,723	4.00%	16,741	21.30%	
80+	10,691	1.60%	8,655	11.00%	
**Type**
Ambulatory	564,547	83.70%	8,977	11.40%	<0.0001
Hospitalization	109,773	16.30%	69,515	88.60%	
**Comorbidities**
Diabetes	85,614	12.70%	30,096	38.30%	<0.0001
COPD	7,085	1.10%	3,813	4.90%	<0.0001
Asthma	17,889	2.70%	1,578	2.00%	<0.0001
Immunocompression	6,159	0.90%	1,935	2.50%	<0.0001
Hypertension	109,956	16.30%	35,221	44.90%	<0.0001
Cardiovascular disease	10,572	1.60%	4,175	5.30%	<0.0001
Obesity	116,071	17.20%	19,182	24.40%	<0.0001
Renal chronic disease	8,635	1.30%	5,529	7.00%	<0.0001
Smoking	48,622	7.20%	6,264	8.00%	<0.0001
**Outcomes**
Pneumonia	79,584	11.80%	58,078	74.00%	<0.0001
Invasive mechanical ventilation	5,898	0.90%	25,539	32.50%	<0.0001
Intensive care unit	7,514	1.10%	7,890	10.10%	<0.0001

*The value of p was calculated by X^2^ test*.

The authors apologize for these errors and state that this does not change the scientific conclusions of the article in any way. The original article has been updated.

## Publisher's Note

All claims expressed in this article are solely those of the authors and do not necessarily represent those of their affiliated organizations, or those of the publisher, the editors and the reviewers. Any product that may be evaluated in this article, or claim that may be made by its manufacturer, is not guaranteed or endorsed by the publisher.

