# Supplementary material for: Coronavirus Disease-2019 Survival in Mexico: A Cohort Study on the Interaction of the Associated Factors
Source: Front Public Health. 2021 Jul 27;9:660114. doi: 10.3389/fpubh.2021.660114 (PMC8353107; doi:10.3389/fpubh.2021.660114)
Supplement: Supplementary file 1 [file Table_1.DOCX]

Supplementary Material: Nelson-Aalen cumulative hazard (left) and kernel density smoothed hazard (right) functions, for inpatients and outpatients.
